# Capture and characterization of antibiotic resistance plasmids from Beargrass Creek, an urban watershed in Louisville, KY

**DOI:** 10.17912/micropub.biology.002240

**Published:** 2026-09-14

**Authors:** Savonna Carrie, Andrew Goeppner, Landon Patel, David S. Treves

**Affiliations:** 1 Biology, IU Southeast

## Abstract

The objective of this work was to capture and characterize antibiotic resistant plasmids from Beargrass Creek in Louisville, KY, an extensive urban watershed that receives combined sewer overflow and surface runoff. Twenty-eight plasmids captured from creek water were screened for antibiotic resistance, surveyed for common antibiotic resistance genes, and subjected to restriction digestion to determine common plasmid backbones. Based on this analysis, five plasmids were chosen for sequencing that revealed TEM-type β-lactamases conferring ampicillin resistance and *tetA* associated with tetracycline resistance. This work provides a better understanding of plasmid borne antibiotic resistance and plasmid diversity in urban watersheds.

**Figure 1. Antibiotic Resistance Plasmids Captured from Beargrass Creek:** Simplified maps of the five plasmids described in this study. Color scheme: red, antibiotic resistance; yellow, mobile genetic elements; green, replication and plasmid maintenance; blue, accessory genes. Images are not proportional to plasmid size. Plasmids A5, A11 and 2-1 encode TEM *-* type beta-lactamases providing resistance to ampicillin/cephalothin; plasmids ST2 and T1 encode *tetA* / *R* providing tetracycline resistance. In addition to antibiotic resistance genes, mobile genetic elements such as *tnpA* , *tnpR* , *insB* and tra2_ECOLX are associated with the five sequenced plasmids.

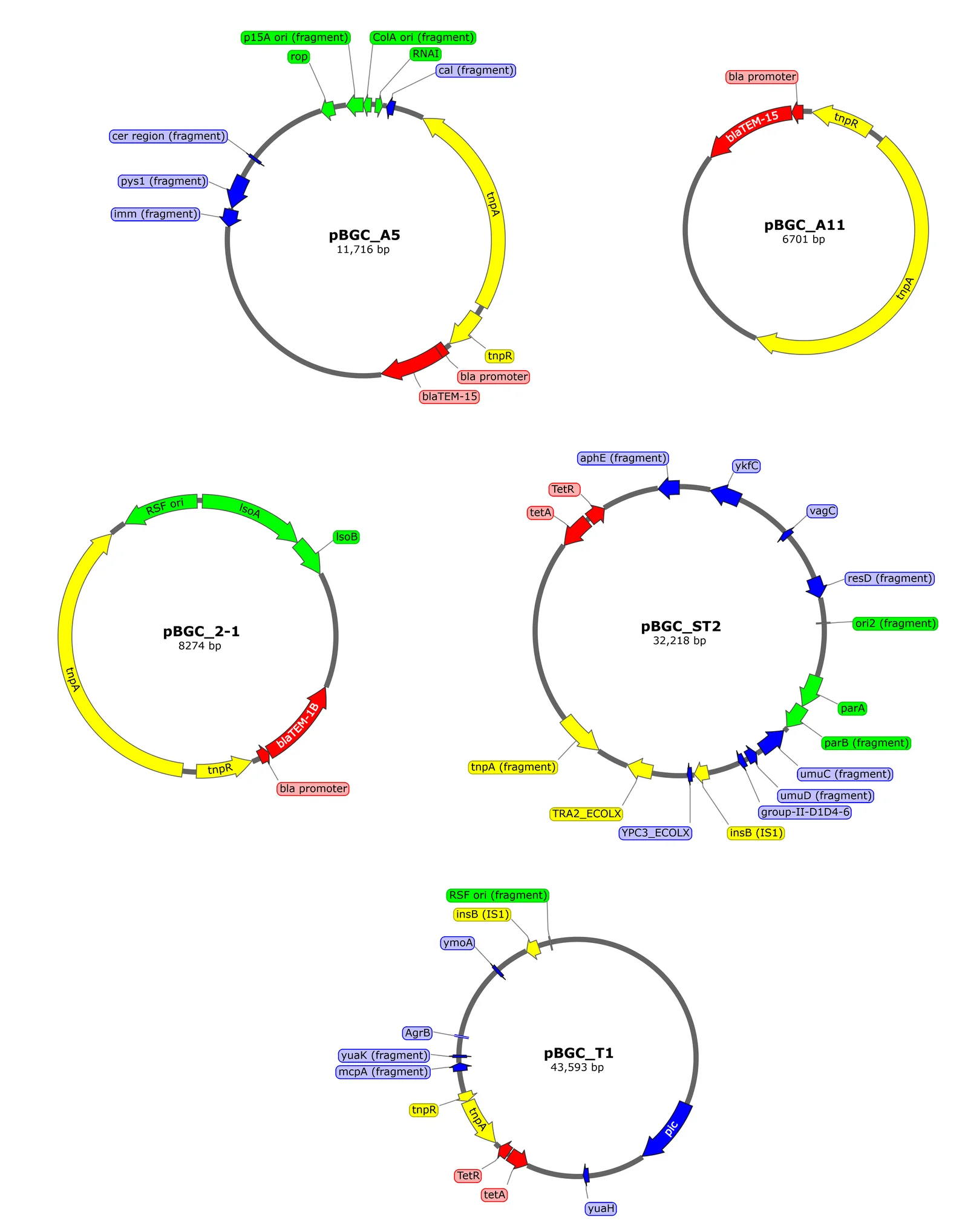



## Description

Antimicrobial resistance (AMR) is a global healthcare crisis with scientists warning of an imminent post-antibiotic era (Carlet et al. 2012, Kwon & Powderly 2021). In 2019 nearly five million deaths were associated with antimicrobial resistant bacterial infections (Murray et al. 2022) and predictions for 2050 set this number at 8.2 million unless interventions are made (Naghavi et al. 2024).

Bacterial plasmids play a key role in the AMR crisis as they carry antibiotic resistance genes. Bacteria often transfer plasmid-borne genes horizontally, and subsequent bacterial movement between humans, animals, and the environment drives wider dissemination of antibiotic resistance (Castañeda-Barba et al. 2024). While there is a large focus on AMR in clinical and agricultural settings, an understanding of antibiotic resistance in the environment may identify resistance patterns and selection pressures that inform AMR trends in humans and animals (Larsson & Flach 2022).

The objective of this study was to isolate and characterize antibiotic resistance plasmids from the Beargrass Creek watershed, an urban stream covering ~60 square miles in Louisville, KY. In addition to surface runoff, this watershed receives untreated sewage from combined sewer overflows and *Escherichia coli* estimates at multiple sampling sites regularly exceed water quality standards (MSD 2022). Screening for antibiotic resistant plasmids in Beargrass Creek is important as urban runoff from combined sewer overflows and surface runoff is a significant source of antibiotic-resistant bacteria (Meradji et al. 2025).

Plasmids from Beargrass Creek were isolated by bacterial enrichment from creek water followed by plasmid extraction. *E. coli* was transformed with extracted plasmid fractions followed by selection for ampicillin or tetracycline resistance. Using this approach, plasmid capture experiments from 2018-2022 yielded eighteen plasmids with ampicillin/cephalothin resistance and ten plasmids with resistance to tetracycline.

Plasmid restriction profiles from the captured plasmids were generated to identify common plasmid backbones, and PCR screening identified *bla* TEM beta-lactamases in the ampicillin/cephalothin resistant plasmids and *tetA* from the tetracycline resistant plasmids. To better understand plasmid structure, five representative plasmids from the collection of captured plasmids were chosen for sequencing using Oxford Nanopore Technology (Figure 1).

The three ampicillin/cephalothin resistant plasmids selected for sequencing ranged in size from 6.7 to 11.7 Kb and all carry *bla* TEM gene variants (Figure 1). TEM-type extended spectrum beta-lactamases (ESBLs) were discovered in the 1960s and there are now over 200 variants (Castanheira et al. 2021). While bacteria with TEM-type ESBLs caused outbreaks in the 1990s, the newer CTX-M ESBL family have since spread worldwide to become the dominant ESBL associated with healthcare and community acquired infections (Doi et al. 2017). The two tetracycline resistant plasmids chosen for sequencing ranged from 32 Kb up to 43 Kb and both carry the *tetA* resistance gene (Figure 1). *tetA* codes for an energy dependent efflux pump that removes tetracycline from the cell (Grossman 2016). Antibiotic efflux, along with ribosome protection, are common tetracycline resistance mechanisms often found in bacterial pathogens (Markley & Wencewicz 2018).

In addition to antibiotic resistance, mobile genetic elements are associated with the five sequenced plasmids. Four of the five plasmids have adjacent *tnpA* transposase and *tnpR* resolvase genes which are common to the Tn3 transposon family (Partridge et al. 2018). Plasmids ST2 and T1 carry the *insB* gene that is essential for IS1 transposition (Escoubas et al. 1991) and plasmid ST2 also carries the probable transposase tra2_ECOLX from transposon Tn903 (Grindley and Joyce 1980).

Sequencing revealed plasmid pBGC_2-1 carries a type II toxin-antitoxin system coded by the *lsoA* and *lsoB* genes. This system, which was discovered on an enterohemorrhagic *E. coli* O157:H7 plasmid, functions both in plasmid persistence and phage defense via degradation of mRNA when the *lsoB* antitoxin is depleted (Otsuka & Yonesaki 2012). Other genes associated with plasmid maintenance include *ROP* / *RNAI* on pBGC_ST2 which controls plasmid copy number (Helmer-Citterich et al. 1988), and *parA* with a *parB* fragment on pBGC_A5 which when functional regulates plasmid partitioning (Friedman and Austin 1988). Although virulence factors were not common in the plasmids characterized in this study, plasmid pBGC_T1 carries the *pic* gene which produces a serine protease autotransporter that promotes mucin degradation and intestinal colonization (Henderson et al. 1999).

While the plasmid capture method described here successfully isolated antibiotic resistance plasmids, future research could apply different plasmid capture techniques and alternative selective conditions to further study plasmid diversity in Beargrass Creek. For example, Botts et al. (2017) used both direct culture (endogenous) and bi-parental mating (exogenous) techniques to isolate plasmids from an urban coastal wetland. Direct culture methods and selection with antibiotic cocktails allowed Botts et al. (2017) to successfully isolate multi-drug resistance plasmids from the wetland environment. Plasmid capture by direct culture allows for identification of the original host species, although this technique may miss plasmids carried by fastidious bacteria.

Exogenous plasmid isolation that relies on mating environmental bacteria to a recipient host is a logical next step to study plasmid diversity in Beargrass Creek. This method does not require purification of the original host bacterium, isolates both conjugative and mobilizable plasmids, and can be used with multiple recipient bacteria (Smalla et al. 2015). Delaney et al. (2018) compared various plasmid capture techniques from cecal broiler samples and found that exogenous isolation provided the best representation of antibiotic-resistant plasmids.

The work described here provides baseline data for understanding plasmid borne antimicrobial resistance in Beargrass Creek. Capture experiment in this study yielded unique plasmids, suggesting that Beargrass Creek still holds undiscovered plasmid diversity. A more complete view of plasmid diversity in Beargrass Creek may be achieved by using different antibiotics (or antibiotic cocktails) for selection, and alternate plasmid isolation techniques such as exogenous plasmid capture. Newly developed plasmid screening techniques of metagenomic libraries, which successfully identified novel plasmids from the human gut microbiome (Camargo 2024), could also be applied to the Beargrass Creek watershed. Future research could determine if certain antibiotic resistance plasmids are established in Beargrass Creek, monitor seasonal dynamics of plasmids in the watershed, and explore plasmid diversity during high water events when contamination from combined sewer overflow is most likely.

## Methods

Site description and sampling: Water samples were collected in sterile polypropylene containers from the middle fork of Beargrass Creek near Big Rock Park in Louisville, Kentucky (38.233145, -85.684123). Samples were transported at ambient temperature to IU Southeast (~30 min) where they were immediately processed.

Enrichment of bacteria: Water samples were filtered onto sterile 0.45 µm membranes (Millipore) and incubated on m-Endo Broth (Hach) for 24 h at 37°C. Cells were rinsed from the membranes with sterile distilled water and plasmid DNA was extracted by alkaline lysis using Promega reagents.

Plasmid capture by transformation: *E. coli* 5-alpha (NEB) or E. cloni (Lucigen) were transformed with Beargrass Creek plasmid fractions following manufacturers guidelines and 50-100 microliter aliquots were plated onto LB plates containing ampicillin (100 µg/mL) or tetracycline (12 µg/mL) followed by incubation at 37°C for 24-48 hours.

Transformant characterization: Potential transformants were screened for antibiotic resistance by disc diffusion (Bauer et al. 1966). PCR of resistance genes used Promega GoTaq Green Master Mix with primers for *blaTEM* (Colom et al. 2003) or *tetA* (Sengeløv et al. 2003).

DNA sequencing: Plasmids were prepared by Zyppy Prep (Zymo) and whole plasmid sequencing was performed by Plasmidsaurus using Oxford Nanopore Technology long read technology ( https://plasmidsaurus.com/ ). Initial plasmid annotation was done with Plannotate (McGuffie & Barrick 2021) followed by Resfinder (Florensa et al. 2022), PlasmidFinder (Carattoli et al. 2014) and RAST (Aziz et al. 2008) to confirm annotation and identify additional unique features.

Data availability: The plasmid nucleotide sequences reported in this study are available in the Genbank database under Bioproject number PRJNA1376510 .
